# Real-Time Motion Analysis Reveals Cell Directionality as an Indicator of Breast Cancer Progression

**DOI:** 10.1371/journal.pone.0058859

**Published:** 2013-03-19

**Authors:** Michael C. Weiger, Vidya Vedham, Christina H. Stuelten, Karen Shou, Mark Herrera, Misako Sato, Wolfgang Losert, Carole A. Parent

**Affiliations:** 1 Laboratory of Cellular and Molecular Biology, Center for Cancer Research, National Cancer Institute, National Institutes of Health, Bethesda, Maryland, United States of America; 2 Department of Physics, University of Maryland, College Park, Maryland, United States of America; 3 Laboratory of Cancer Biology and Genetics, Center for Cancer Research, National Cancer Institute, National Institutes of Health, Bethesda, Maryland, United States of America; Wayne State University School of Medicine, United States of America

## Abstract

Cancer cells alter their migratory properties during tumor progression to invade surrounding tissues and metastasize to distant sites. However, it remains unclear how migratory behaviors differ between tumor cells of different malignancy and whether these migratory behaviors can be utilized to assess the malignant potential of tumor cells. Here, we analyzed the migratory behaviors of cell lines representing different stages of breast cancer progression using conventional migration assays or time-lapse imaging and particle image velocimetry (PIV) to capture migration dynamics. We find that the number of migrating cells in transwell assays, and the distance and speed of migration in unconstrained 2D assays, show no correlation with malignant potential. However, the directionality of cell motion during 2D migration nicely distinguishes benign and tumorigenic cell lines, with tumorigenic cell lines harboring less directed, more random motion. Furthermore, the migratory behaviors of epithelial sheets observed under basal conditions and in response to stimulation with epidermal growth factor (EGF) or lysophosphatitic acid (LPA) are distinct for each cell line with regard to cell speed, directionality, and spatiotemporal motion patterns. Surprisingly, treatment with LPA promotes a more cohesive, directional sheet movement in lung colony forming MCF10CA1a cells compared to basal conditions or EGF stimulation, implying that the LPA signaling pathway may alter the invasive potential of MCF10CA1a cells. Together, our findings identify cell directionality as a promising indicator for assessing the tumorigenic potential of breast cancer cell lines and show that LPA induces more cohesive motility in a subset of metastatic breast cancer cells.

## Introduction

Cell motility is essential during development, wound healing and immune responses, and plays a prominent role during pathological conditions such as tumor invasion and metastasis [1], [2]. As cancer progresses, tumor cells invade surrounding tissues and metastasize to distant sites. Metastasis is a major cause of mortality among cancer patients, especially in individuals diagnosed with breast cancer [3], [4], [5]. Invasive and metastatic tumor cells have altered genetic profiles with deregulated intrinsic signaling cascades, which in turn support both invasive migratory behaviors as well as unregulated growth and survival in heterotopic environments [6], [7], [8]. Furthermore, tumor cells are exposed to a continually evolving extracellular environment both during cancer progression as well as during their migration to metastatic sites. Numerous extracellular signaling molecules are implicated in promoting invasive tumor cell migration including hepatocyte growth factor (HGF), transforming growth factor β (TGF-β), epidermal growth factor (EGF), and lysophosphatidic acid (LPA) [9], [10]. In breast cancer, EGF, which binds to the ErbB receptor tyrosine kinase family [11], [12], has been shown to play a role in the invasion and metastasis of breast cancer [13], [14], [15]. Overexpression of ErbB receptors or HER2/neu increases cancer cell motility and metastasis and is a common feature in many breast cancers [16], [17], [18], [19], [20]. The small phospholipid LPA, which binds to the LPA receptor (LPAR) family of G protein-coupled receptors and couples with at least three G-protein subtypes (G_i_, G_q_, and G_12/13_), has also been reported to modulate epithelial cell migration [21] and enhance the metastatic potential of breast cancer cells [22], [23], [24], [25]. Yet, it remains largely unclear how these intrinsic and extrinsic factors collude to alter cell migration properties during breast cancer progression.

The MCF10A cell series is a breast cancer progression model composed of well-characterized human breast cancer cell lines [26], [27], [28]. The series was established from immortalized mammary epithelial MCF10A cells, which were derived from a patient with fibrocystic disease [29]. The MCF10A cells were transformed with Ras to generate the pre-malignant MCF10At.1k cell line that forms benign hyperplastic lesions after introduction into immune compromised mice. Subsequent passage of MCF10At.1k cells through mice led to the isolation of tumorigenic MCF10CA1h cells and invasive, lung colony-forming MCF10CA1a cells, both of which give rise to tumors within days of introduction into mice [28], [30], [31]. These two tumorigenic cell lines, CA1a and CA1h, harbor an activating mutation in *PIK3CA*, which is mutated in ∼30% of human breast cancer cases [32], [33]. Thus, the MCF10A series represents an advantageous model for assessing how intrinsic mutations associated with breast cancer progression alter migration profiles in cells with a similar genetic background.

An important question remains: can *in vitro* cell migration properties be used as a robust indicator of tumorigenic potential? It is a common practice to assess the migratory potential of tumor cells, which is intricately linked to invasion and metastasis, with transwell or unconstrained 2D migration assays. However, the predictive value of these assays is controversial, particularly if cell lines with different genetic background are compared. More recently, time-lapse imaging and subsequent image analysis using particle image velocimetry (PIV) to quantify cell motion have provided a more revealing view of collective cell migration, especially in the context of unconstrained migration assays [34], [35], [36], [37]. Such methodologies will greatly expand our understanding of how both intrinsic and extrinsic factors contribute to invasive migratory behaviors observed in many cancers, including breast cancer.

In this report, we analyzed the migration of cell lines of the MCF10A series using timelapse imaging coupled with PIV analysis and compared these dynamic measurements to traditional transwell and unconstrained 2D migration assays. Dynamic measurements and quantitative analysis provided high-content information and identified cell directionality as an indicator of tumorigenicity in the breast cancer cell lines analyzed. Additionally, we found that LPA stimulation of cell sheets increases the directionality of movement of metastatic breast cancer cells, suggesting that LPA may have a key role on the metastatic potential of these cells.

## Results

### End point migration assays do not correlate with the malignant potential of breast cancer cell lines

**Figure 1:**
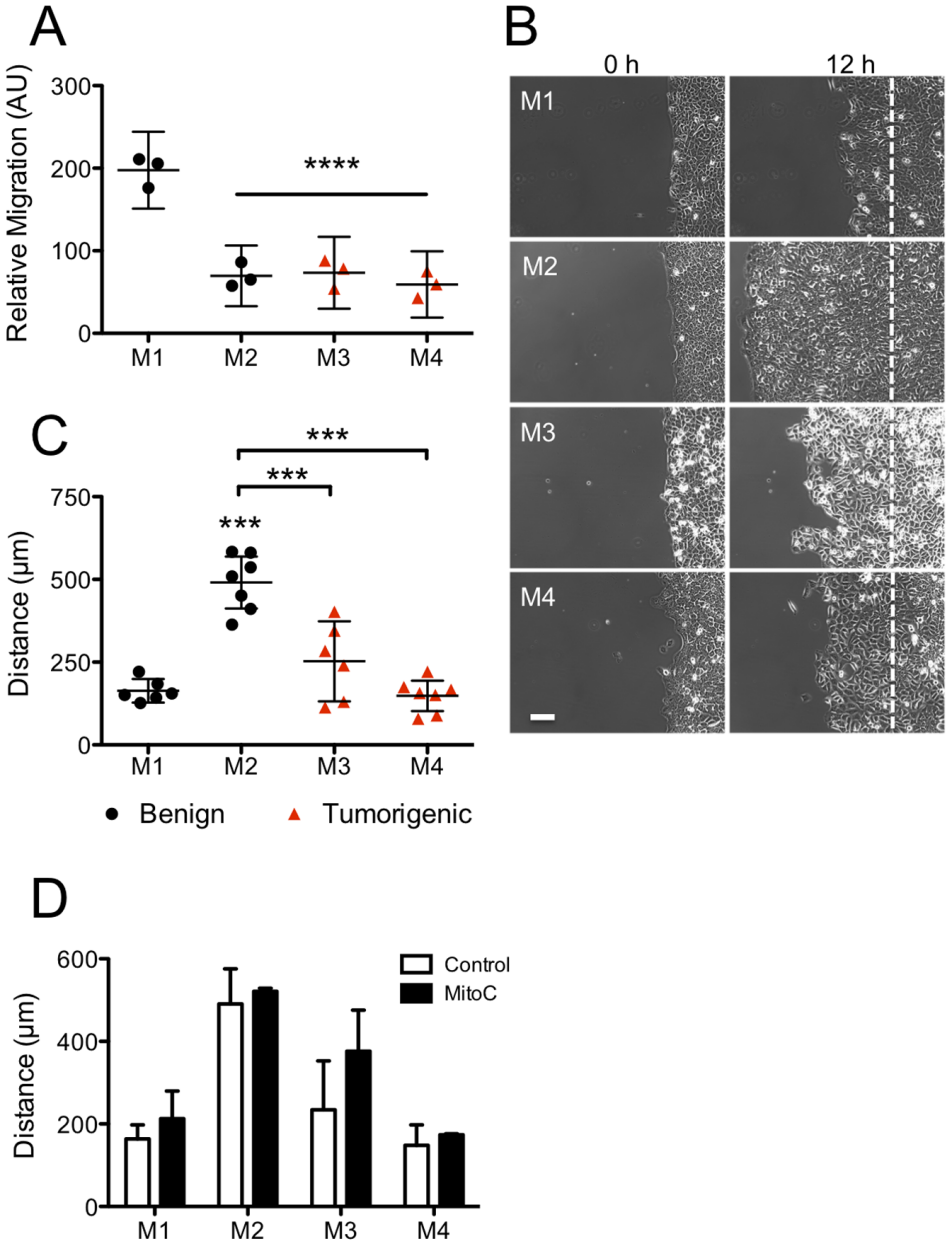
Cell lines of the MCF10A series show distinct migration properties. (**A**) Migration potential of M1–M2 (benign, black circles) and M3–M4 (tumorigenic, red triangles) cell lines after 4 h was assessed with the transwell assay using collagen IV coated membranes and no biased stimulation (see also Fig. S1A). (**B**) Phase images of the M1–M4 cell lines after 0 and 12 h of unconstrained migration. The dash vertical line indicates the initial location of the sheet edge. Scale bar  = 100 µm. (**C**) Quantification of the net displacement (during the 3–12 h time frame) is presented as in panel A. (**D**) M1–M4 cells were first pretreated with 25 µg/mL Mitomycin C for 20 min and then allowed to migrate into open space under conditions identical to panel B (black bars). The net displacement (mean ± SD) is shown compared to control (w/o drug) conditions (white bars), n = 2. For panels A and C results are presented as mean ±95% CI of 6–7 independent experiments. Statistical significance: * p<0.05, ** p<0.01, *** p<0.001, ****p<0.0001 (Tukey-Kramer test, n = 6–7). All comparisons were made with M1 cells unless indicated by pairing-brackets.

To study alteration of cell migration during breast cancer progression, we used cell lines of the MCF10A series. For simplicity, the cell lines will be referred to as follows: epithelial MCF10A (M1), Ras-transformed, premalignant MCF10At.1k (M2), tumorigenic MCF10CA1h (M3), invasive, lung-colony forming MCF10CA1 (M4), which we further divided into benign (M1 and M2) and tumorigenic (M3 and M4) [28], [29], [30], [31]. We first assessed the migratory capacity of each cell line using transwell and unconstrained migration formats [38]. Both formats take end-point measures of either the number of cells migrating across a porous membrane (for the transwell assay) or the distance migrated (for the unconstrained migration assay). Using the transwell assay coated with collagen IV we found that the basal-intrinsic migration ability of the cell lines varied, with M1 cells migrating much more efficiently than the other cell lines, including the invasive M4 cell line (Fig. 1A and Fig. S1A).

Next we assessed the migration capacity of the MCF10A series using the unconstrained migration assay. Briefly, cells were plated in a tissue culture insert on collagen IV coated 12-well glass bottom dishes. After overnight culture, the insert was removed and migration of the epithelial sheet into open space under basal conditions was assessed 12 h later (Fig. S1B). We found that the average displacement of the epithelial edge is distinct for each cell line (Fig. 1B, C), M2 cells having the greatest net displacement of the epithelial edge, followed by M3 cells. The epithelial edge of M1 cells and, remarkably, M4 cells show the least net displacement. To confirm that cell proliferation was not significantly contributing to the net displacement observed for each cell line, we pretreated cells with Mitomycin C and found that inhibiting cell proliferation has minimal impact on the net displacement in all four cell lines (Fig. 1D). Additionally, the number of mitotic events across cell lines showed little correlation with the net displacement of their epithelial edge in the absence of Mitomycin C (Fig S2A). For example, while M2 cells had the highest rate of mitotic events and covered the greatest distance, M4 cells had a high proliferation rate, but traveled a much shorter distance. Although cell proliferation has a role in maintaining epithelial sheet density for migration [36], [39], proliferation does not drive the epithelial edge net displacement observed in the unconstrained migration assays used here.

Taken together, these data indicate that under basal conditions the transwell and unconstrained migration assays are not interchangeable for the breast cancer cells studied here. Furthermore, both assays fail to distinguish tumorigenic from benign cell lines and reveal little about how breast cancer progression impacts collective motility.

### Cell lines of the MCF10A series have distinct migration speed and directionality

We then set out to more comprehensively examine the migration properties of the MCF10A series by coupling live cell, time-lapse imaging with the PIV technique to quantitatively analyze cell motion. From the time-lapse recordings of the unconstrained migration assays, it became clear that the cell lines of the MCF10A series have distinct morphologies and motion dynamics (Movies S1, S2, S3, S4). We found that all cell lines show a persistent outward migration for 12 hrs after an initial lag phase of ∼3 hrs. While M1 and M2 cells typically move as tight epithelial sheets almost perpendicular to the initial constraining boundary, M3 and M4 cells move more independently and form swirling clusters that occasionally brake away from the edge of the cell sheet.

**Figure 2:**
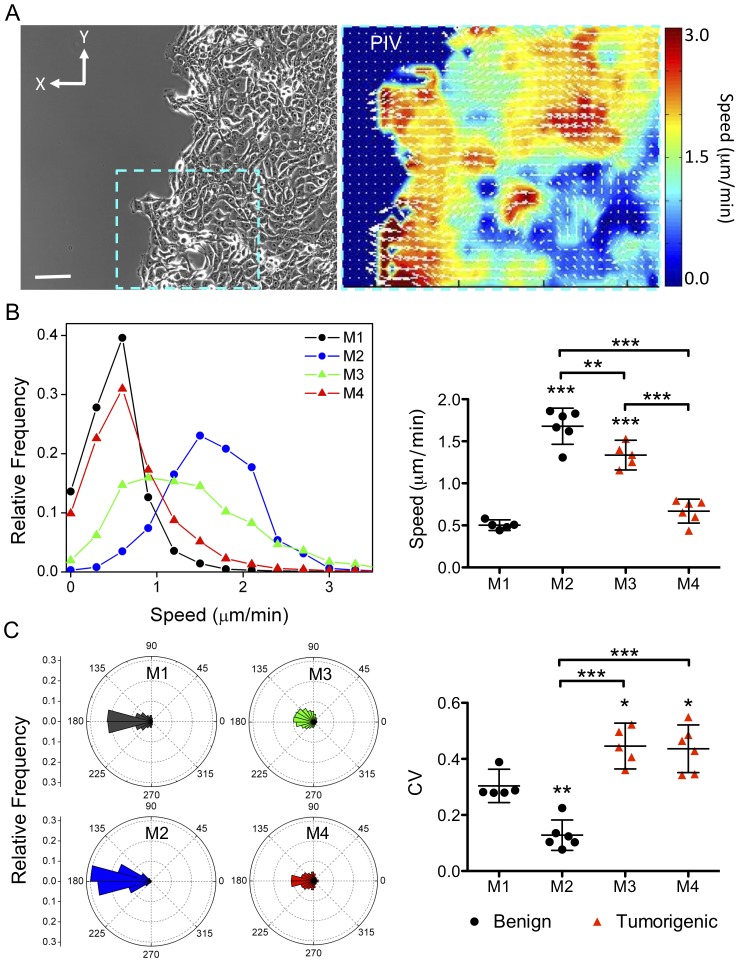
Cell lines of the MCF10A series show distinct migration speed and directionality. (**A**) PIV analysis enables the mapping of velocity fields associated with the underlying epithelial sheet motions captured by phase time-lapse imaging (scale bar  = 100 µm). Spatial profiles of directionality and speed are depicted with white vectors and a heat map, respectively (right panel). (**B**) Left: Aggregate speed distributions, determined over all times and space, were compiled from 5–6 independent experiments for each cell line. Right: Quantification of the mean of the average speed (mean ±95% CI) for each cell line; M1–M2 (benign, black circles) and M3–M4 (tumorigenic, red triangles). (**C**) Left: Rose plots depicting aggregate directionality distributions were compiled over all times and space for each cell line (n = 5–6). Right: Variability of the direction of motion was quantified by the coefficient of variation (CV) and reported as mean ±95% CI. Statistical significance: * p<0.05, ** p<0.01, *** p<0.001 (Tukey-Kramer test, n = 5–6). All comparisons were made with M1 cells unless indicated by pairing-brackets.

In order to analyze these migration patterns in a quantitative manner, we employed PIV to obtain the velocity fields of the epithelial sheets. Briefly, PIV analysis divides each acquired image (frame) into small (32×32 pixel) interrogation regions and finds the most similar interrogation region in a nearby location in the next frame. The shift of the interrogation region indicates the speed and direction of motion of this region in the image. As the time interval between frames is known, a velocity map that describes the motion of the epithelial sheet over time can be generated, and speed and directionality of motion in the cell sheet can be calculated (Fig. 2A). We found that M2 and M3 cells have very broad speed distributions (Fig. 2B, left panel). Interestingly, M4 cells exhibit a much narrower speed distribution that is similar to M1 cells, even though the average speed of M4 cells is slightly higher than that of M1 cells. Furthermore, the mean x-directional speed for each cell line (Fig. S2B) mirrors the net displacement of the epithelial edge (Fig. 1C), confirming our initial results and indicating that PIV can faithfully capture this end-point measurement. Indeed, we find that average speed measurements are also not indicative of the tumorigenic potential of the cell lines (Fig. 2B, right panel).

Next, we analyzed the directionality of movement (or angle of motion) in the cell sheet. Comparisons of both angular distributions (Fig. 2C, left panel) and coefficients of variation in directionality (CV; Fig. 2C, right panel) identify a measure of tumorigenic potential for these cell lines. Indeed, we found that tumorigenic M3 and M4 cells display significantly less directed motion than the non-transformed M1 and pre-malignant M2 cells (Fig. 2C, right panel). Taken together a comprehensive analysis of cell motility indicates that directionality of cell movement is markedly suppressed in these tumorigenic breast cancer cell lines compared to their benign counterparts.

### Cell lines of the MCF10A series show distinct responses to EGF and LPA

We next investigated the effect of EGF and LPA, two key effectors of cell migration [40], [41], [42], on the migratory properties of the MCF10A series. First we assessed the expression profile of key signaling components in the cell lines. All four cell lines express EGFR and various LPARs (Fig. S3A & B) as well as Erk and Akt, which are involved in the intracellular transduction of these signals. Under basal conditions, M2, M3, and M4 cells have high levels of phosphorylated Erk. Additionally, M3 and M4 cells exhibit elevated phosphorylated Akt levels, consistent with previous reports [27]. Both, EGF and LPA treatments increase the phosphorylation of Erk and Akt in M2 cells, while only M1 cells show increased phosphorylation of Erk after 6 hrs of EGF stimulation. EGF and LPA do not significantly alter cell proliferation in any of the cell lines (Fig. S2C).

**Figure 3:**
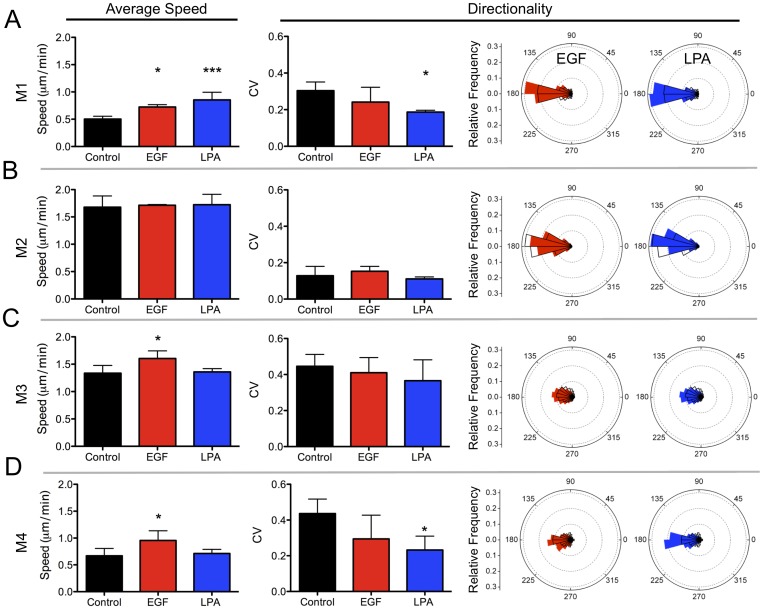
Cell lines of the MCF10A series show distinct responses to EGF and LPA. (**A–D**) M1–M4 cells were stimulated with 5 ng/mL EGF (red) or 1 µM LPA (blue) and perturbations of average speed and of directionality (angle distributions and CV) compared to controls (black) were assessed (mean ± SD). Rose plots depict controls (unfilled, black bars) and 5 ng/mL EGF (filled, red bars) or 1 µM LPA (filled, blue bars). Statistical significance: * p<0.05, ** p<0.01, *** p<0.001 (Tukey-Kramer test, n = 3 for all conditions except M2 with EGF where n = 2). All comparisons were made with M1 cells unless indicated by pairing-brackets.

While assessing the migration behavior, we found that stimulation with EGF (5ng/ml) increases the average speed of M1, M3 and M4 cells without significantly affecting directional movement in any cell line (Fig. 3A,C & D). In comparison, LPA dramatically increases directional movement in M1 and M4 cells, while only increasing speed in M1 cells (Fig. 3A & D). M2 cells are insensitive to either EGF or LPA (Fig. 3B). Interestingly, the suppression of random movement in M4 cells following LPA treatment allows this metastatic cell line to adopt a migratory phenotype similar to that of un-stimulated, non-transformed M1 cells (Movies S1 & S5). Together, these data show that EGF and LPA have varying effects on the migration properties of the cell lines of the MCF10A series. While EGF appears to enhance chemokinetic behaviors characterized by increased migratory speeds with little or negative effects on directed motion, we found that LPA stimulation increases directionality in M1 and M4 cells, but only increases cell speed in M1 cells.

### Cell lines of the MCF10A series display distinct spatiotemporal speed and directionality patterns during collective motion

**Figure 4:**
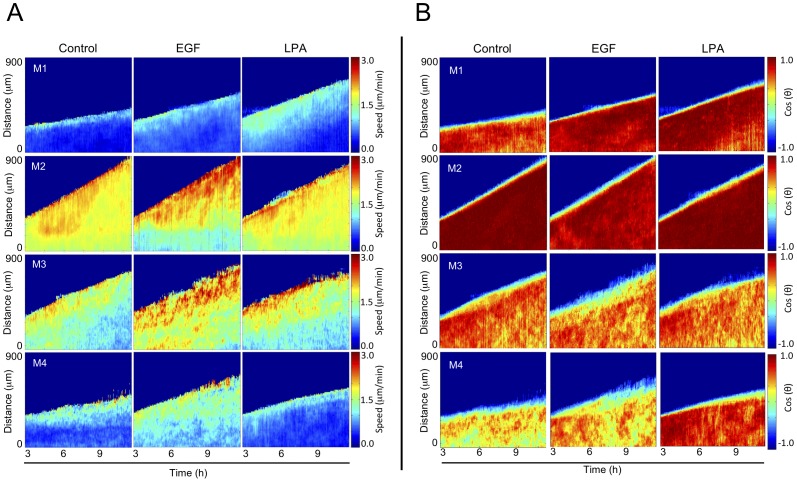
Cell lines of the MCF10A series display distinct spatiotemporal speed and directionality patterns during collective motion. Spatiotemporal heat plots show the average (A) speed and (B) directionality as a function of both position and time from the edge to the center for the sheet. This yielded a spatial map of average speeds and directionalities starting at the sheet edge and moving away, toward the inner regions of the sheet. We defined cos (180°) = 1 (motion directed toward open space) and cos (0°) = −1 (motion directed away from open space). Since cell movement is minimal during the first 3 h, spatiotemporal plots were generated between 3 and 12 h. Cells were stimulated with buffer (control), EGF (5 ng/ml) or LPA (1 µM). Data show heat plots calculated from representative experiments (n = 3 for all conditions except M2 with EGF where n = 2).

Time-lapse imaging of migrating M1–M4 cells established that the migration pattern of cells varies within the sheet, with distinct cellular behaviors along the edge of the epithelial sheet compared to cells located more towards the center of the sheet (Movies S1, S2, S3, S4). We therefore analyzed the speed and directionality of the epithelial sheet as a function of the position within the sheet over time and created spatiotemporal heat plots to illustrate the differences among cell lines. We found that under basal conditions, regions near the epithelial edge generally move faster (∼3 µm/min) than areas towards the center of the sheet (<2 µm/min). In addition, M2 cells, and to a lesser extent M3 cells, show a broader area near the epithelial edge of highly active regions compared to M1 and M4 cells (Fig. 4A). As expected, M2 and M3 cells are faster than M1 or M4 cells. Importantly, M3 and M4 cells exhibit very heterogeneous, speed and directionality profiles compared to M1 and M2 cells, which show a remarkably homogenous directionality toward the open space (Fig. 4A & B). This further confirms our finding that tumorigenic cells (M3 and M4) exhibit considerable differences in their direction of motion compared to the two benign cell lines (M1 and M2) and underscores the relevance of live cell imaging methods that capture and measure cell migration dynamics.

We were also able to more clearly show that EGF stimulation regulates speeds throughout the cell sheet for each cell line. We found that regions near the front of the epithelial sheet are faster compared to basal conditions, except for M2 cells, which move slower in regions far from the epithelial edge (Fig. 4A). Consistent with this, we did not measure an increase in the average speed of M2 cells (Fig. 3B, left panel). In addition, as our average measures of both angular distributions and coefficients of variation in directionality showed (Fig. 2), we found that EGF treatment does not alter the spatiotemporal directionality profile of any of the cell lines (Fig. 4B). In contrast, LPA treatment specifically modifies the migration patterns of M4 and M1 cells (Fig. 4A). In M1 cells, LPA broadens and increases the speed of the epithelial edge. In M4 cells, LPA reverts the highly heterogeneous spatiotemporal speed and directionality distribution to homogeneous distributions that mirror those observed in un-stimulated M1 cells (Fig. 4A&B). We further found that pretreatment of M4 cells with the LPA1 and LPA3 receptors antagonist Kil6425 [43] inhibits the effect of LPA on the M4 cells (Fig. S4A). Taken together, our findings show that breast cancer cell lines of increasing malignancy exhibit progressively more heterogeneous spatiotemporal speed and directionality profiles that can be differentially perturbed by EGF or LPA. While EGF generally increases cell speed, LPA selectively reduces spatiotemporal heterogeneity in the metastatic M4 cells.

The heterogeneous speed and directionality distributions observed in the M3 and M4 cell lines suggest that these cells have lost their epithelial character and perhaps have undergone an epithelial-mesenchymal transition (EMT). To investigate this further we measured the relative levels and spatial localization of E-cadherin and vimentin using immunofluorescence. We find that M3 cells show dramatically reduced E-cadherin staining compared to M1, M2, and M4 cells (Fig. S6) and we confirmed low expression levels in Western blots (Fig. S7). Interestingly, M4 cells still express E-cadherin, albeit at lower levels compared to M1 cells (Fig. S6 & S7). Although all cell lines express vimentin, in M1 and M4 cell sheets we observe varying expression levels of vimentin in cells at the edge of the sheet, whereas in M2 and M3 cells sheets vimentin expression does not vary much (Fig. S8). EGF or LPA stimulation did not robustly alter E-cadherin and vimentin staining levels and localization patterns in any of the cell lines. Although EMT may contribute to the alterations in migratory properties we observe in these cells, the absence of changes in EMT markers in response to EGF or LPA suggests that other pathways involved in cell migration are also important.

### The migration properties of metastatic MDA-MB 231T cells resemble that of lung colony-forming M4 cells

**Figure 5:**
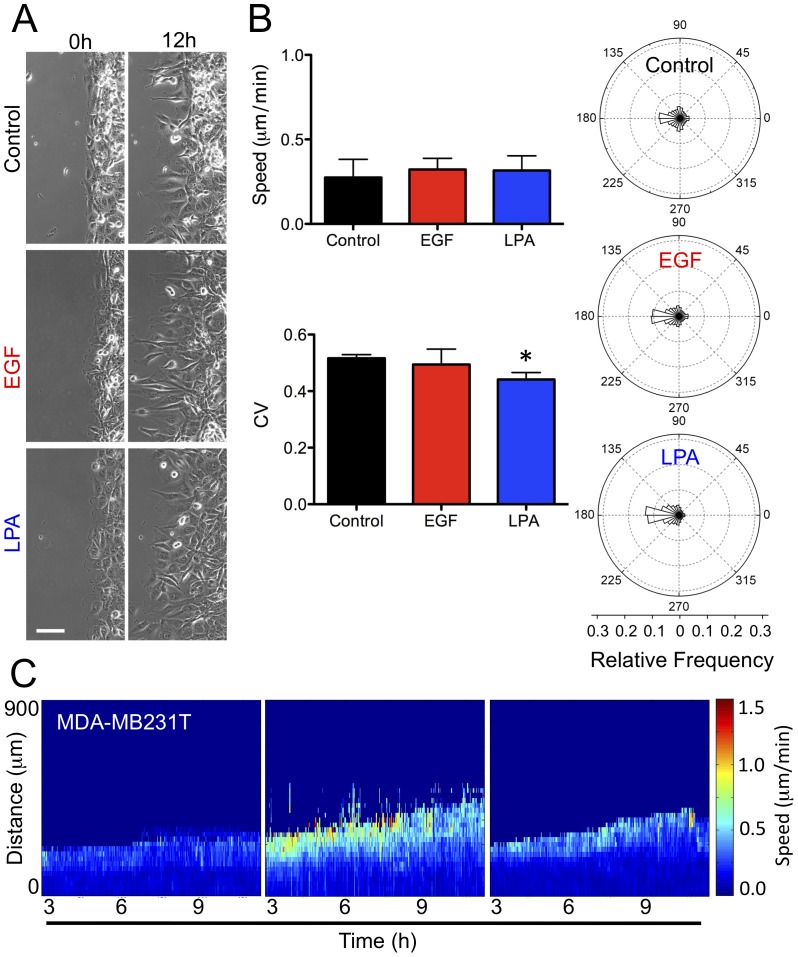
The migratory phenotype of MDA-MB 231T is similar to that of M4 cells. (**A**) Phase contrast images of MDA-MB-231T cells moving into open space after 12 h under control (Black), 5 ng/mL EGF (red), and 1 µM LPA (blue) treatments. Bar = 100 µm. (**B**) Right: The effects of EGF and LPA treatment on average speed (top) and directionality, CV (bottom) determined over all times and space, were compiled from 5–6 independent experiments and reported as mean ± SD. Left: Aggregate directionality profiles for control, EGF and LPA conditions. Statistical significance: *p<0.05 (Tukey-Kramer test, n = 3). (**C**) Representative spatiotemporal heat plots show speed responses in control, EGF, and LPA treated cells. See Fig. 4 for details.

We next set out to determine if the migration properties we identified in the MCF10A series, in particular in the M4 cells, are conserved in another cell line, MDA-MB-231T [44]. MDA-MB-231T breast cancer cells and MCF10A-derived cell lines share the same classification (basal B) and like M4 cells, MDA-MB-231T form lung colonies [44], [45]. Unlike the cells in the MCF10A series, MDA-MB-231T cells exhibit a distinct mesenchymal appearance (Fig. 5A) and migrate at a much slower average speed than even the M4 cells (0.27±0.11 µm/min and 0.67±0.14 µm/min, respectively) (Fig. 5B and Fig. 3D). Nevertheless, like the other tumorigenic cell lines (M3 and M4), the MDA-MB-231T cells exhibit similar levels of random motion, reflected in both angular distribution and CV (Fig. 5B). This result further bolsters our earlier finding that directionality is an indicator for tumorigenic potential under basal conditions, as compared to other measures (Fig. S5A, B, and C). In addition, we found that exogenous stimulation with either EGF or LPA does not increase the average speed of MDA-MB-231T cells, but, much like in M4 cells, LPA significantly enhances directionality of the MDA-MB-231T cells (Fig. 5B). Similarly, the spatiotemporal speed and directionality profiles resemble those of the M4 cells (Fig. 5C & S4B). Notably, our analysis again identified LPA as an exogenous molecule capable of altering the directionality of a metastatic, breast cancer cell line without increasing cell speed.

## Discussion

The acquisition of aggressive migration behaviors is critical during cancer progression and metastasis. In tumors, amoeboid, mesenchymal, chain or collective group migratory behaviors have been observed [46], [47], [48], [49] and the need for new methodologies to investigate the intrinsic and extrinsic factors that regulate these modes of migration is essential. The dynamic nature of cell migration argues for techniques that are able to capture and quantify these dynamics in a way that is easily attainable and comparable between studies. While *in vivo* and 3D experimental assays best recapitulate the environmental conditions present in the body, these methods are technically challenging and are often incompatible with traditional biochemical assays [50]. Furthermore, the majority of today's screening techniques to identify molecular regulators of cell migration (functional genomics, small molecule drug targeting, etc.) rely on 2D formats that easily lend themselves to conventional microscopy-based analyses [51], [52], [53].

In this study, we assessed the migration potential of the MCF10A series, a model of human breast cancer progression. We found that end-point measurements from transwell or unconstrained migration assays do not provide reliable measures of tumorigenic potential, showing no correlation between the number of cells migrating or the distance migrated and the *in vivo* behavior of the cell lines (i.e. benign or tumorigenic). In contrast, by combining live-cell imaging and PIV to systematically analyze the dynamic migratory properties of breast cancer cells we identified cell directionality as an indicator of tumorigenic potential during breast cancer progression. In general, we observed decreasing 2D cell directionality with increasing *in vivo* tumorigenic potential. Indeed, recent studies also identified correlations between growth factor stimulated 2D migratory behavior (lamellipodial protrusion dynamics) and migratory potential in 3D as well as *in vivo* [54], [55]. In our system, it appears that cancer progression alone (in the absence of extrinsic cues) is sufficient to detectably alter the migratory properties of mammary epithelial cells. How these properties are impacted by extracellular signals may reveal further insights into metastatic disease.

Exogenous factors like EGF and LPA have been widely implicated as promoters of breast cancer cell invasion and metastasis by regulating signals that control cell motility [18], [56]. In our 2D collective migration assay, we observed that the addition of EGF generally leads to increases in cell speed without affecting directionality, thereby acting in a more chemokinetic fashion. Indeed, EGF has previously been reported to increase the motility of several breast cancer cell types that express or over-express ErbB receptors [18], [57], [58], [59]. On the other hand, we found that LPA dramatically increases directionality and cohesiveness of collective movement in M4 and MDA-MB-231T cells. LPA has been shown to increase cell migration in several breast cancer lines, including MDA-MB-231 cells, in transwell and invasion assays [40], [56]. In addition, Boucharaba and colleagues have shown that LPARs are involved in metastasis of breast cancer cells to bones [24], [60]. Our findings now lend insight into the motility mechanism that underlies the effects of LPA on breast cancer metastasis. We show that LPA treatment promotes M4 and MDA-MB-231T cells to adopt a more cohesive and ordered migratory phenotype similar to that observed in untreated M1 cells. Yet, M4 cells are intrinsically very different than M1 cells, harboring specific genetic alterations that promote invasive and metastatic behavior [26], [27]. It remains unclear whether the LPA-mediated suppression of random and erratic motions in the tumorigenic cell lines tested here inhibits or promotes their metastatic potential. LPA might act like a signaling ‘switch,’ changing the mode of cancer cell migration from independent to more collective behavior, in a manner similar but opposite to that recently reported for TGF-β [61]. From the experiments reported here, it is not clear whether the LPA-induced collective, epithelial-like motility behavior inhibits metastasis or whether the suppression of random motions in conjunction with other intrinsic factors, for example metalloprotease activity [26] and constitutive PI3K signaling [27], promote collective invasion and metastasis. It was indeed recently reported that tumor cells with a strong epithelial phenotype are more prone to colonize lungs and bone in prostate and bladder cancer models [62]. Nevertheless, as LPA signaling involves several receptors and at least three G-protein subtypes, it is likely that the effects of LPA on a specific cell will depend on the expression levels of these proteins as well as which downstream signaling pathways are preferentially activated [63]. Indeed, LPA was also recently reported to act as a suppressor of invasive behavior in prostate and pancreatic cancers [64], [65], implying that LPA, similar to TGF-β, may play a dual role in tumor progression [66]. Additional investigations are required to assess the role of LPA during migration of metastatic breast cancer cells and to identify the downstream signaling pathways being activated.

In summary, we describe a novel, multifactorial approach to quantitatively analyze the migratory behaviors in a model of progressively malignant breast cancer cell lines. We demonstrate that a detailed analysis of migration dynamics can identify migration behaviors of breast cancer cells that are missed in studies using traditional end-point measurements. Importantly, we show that in the absence of extrinsic factors, tumorigenic *in vivo* behavior correlates with increased random motion or low directionality in the breast cancer cell lines analyzed. We further show that LPA, but not EGF, distinctly impacts directionality in both the metastatic (M4 and MDA-MB-231T) and ‘normal’ epithelial (M1) cells. As cell motility is a pre-requirement for tissue invasion and metastasis, more detailed analyses of tumor cell migration dynamics with techniques such as PIV, both in response to intrinsic changes and extrinsic cues, will promote the identification of molecular targets to prevent or limit invasive and metastatic tumor disease.

## Methods

### Cell Lines

We used a set of four progressively malignant human breast cancer cell lines: normal, immortal MCF10A cells, premalignant MCF10At1k.cl2 cells, tumorigenic MCF10CA1h cells, and invasive, lung colony forming MCF10CA1a cells (Barbara Ann Karmanos Cancer Institute, Detroit, MI). Cells were grown in DMEM/F-12 media supplemented with 5% horse serum and 1% penicillin and streptomycin (all Invitrogen, Carlsbad, CA). Media for M1 and M2 cells were additionally supplemented with 10 µg/ml insulin (Invitrogen), 10 ng/ml EGF (Peprotech, Rocky Hill, NJ), 0.5 µg/ml hydrocortisone and 100 ng/ml cholera toxin (both Sigma, St. Louis, MO). Cells were kept at 37°C, 5% CO_2_ in a humidified atmosphere, and passaged twice weekly. MDA-MB 231T cells were a kind gift from Patricia Steeg [44] and cultured in DMEM supplemented with 5% fetal calf serum (both from Invitrogen).

### Transwell Assay

To measure intrinsic migration potential through a matrix barrier, cells were detached with a brief trypsin treatment, washed and resuspended (5×10^5^ cells/mL) in base medium containing 0.1% horse serum. 100 µL of the cell suspension was then plated onto 6.5 mm diameter (8 µm pore size, polycarbonate membrane) tissue culture inserts (Corning, Tewksbury, MA) that had been treated with 66 µg/mL of Collagen IV (BD, San Jose, CA). 0.1% horse serum containing medium (DMEM/F12) was also added to the lower chamber to insure no chemical bias for migration and cells were allowed to migrate for 4 hours. Cells were then fixed and stained with DAPI. Cells in the upper chamber were removed, those in the lower side were imaged and quantified. For each of the 3 independent experiments conducted and 4 cell lines (M1–M4), triplicate runs were preformed and five 10× fields were acquired. ImageJ was then used to count the number of stained nuclei above background and greater than 50 pixels^2^.

### Unconstrained Migration Assay

Migration assays were performed in 12 well glass bottom plates coated with collagen IV (10 µg/ml) overnight, washed with PBS and air-dried in a sterile environment. Ibidi wound healing inserts (Ibidi, Verona, MI) were placed in the wells, cells suspended in their respective growth media (0.5×10^6^ cells/ml) and 75 µL of cell suspension were added to each well of the insert (growth area: 0.22 cm^2^). The plates were incubated 37°C, 5% CO_2_ in a humidified atmosphere overnight. Cells were then cultured with serum-free DMEM Advance (Invitrogen) for 5 h, before they were stimulated with 0.1% serum, 0.1% serum and EGF (5 ng/ml), or 0.1% serum and 1 µM LPA (Sigma Aldrich, St. Louis, MO); horse serum was used for M1, M2, M3, and M4 cells; fetal calf serum for MDA-MB-231T cells) and transferred into the incubator chamber of the microscope for time lapse imaging. For Mitomycin C experiments, cells were prepared as described above, except that they were pre-treated with 25 µg/mL Mitomycin C after starvation and then following removal of the ibidi insert treated with 0.1% serum, 0.1% serum and EGF (5 ng/ml), or 0.1% serum and 1 µM LPA.

### Time Lapse Imaging

Stimulated cultures were kept in an incubator microscope (Zeiss Observer.Z1, Zeiss, Goettingen Germany) at 5% CO2, 37°C, in a humidified atmosphere. Phase contrast images of cells were taken every 2 min for 20 hrs using a 10× objective.

### Image Processing

Due to the sensitivity of particle image velocimetry, the motions of intracellular trafficking events and even retrograde flow events were often captured in our phase images. In order to focus our analysis on the translational motions of cancer cells and minimize computational time, we used ImageJ 1.45 s (Wayne Rasband, NIH http://imagej.nih.gov) to process all images in the following manner. The intensities of all images in a sequence were normalized, followed by applying a Gaussian blur filter and minimum filter. Background subtraction, followed by edge detection operations were then used to minimize the non-cell pixel intensities. A final background subtraction was preformed to reduce these intensities to ∼ zero to further enhance computation times.

### Particle Image Velocimetry (PIV)

The PIV analysis was performed using a customized version of the mpiv MATLAB toolbox (MATLAB Central File Exchange: http://www.mathworks.com/matlabcentral/fileexchange/2411-mpiv, BSD License). We used 32×32-pixel interrogation windows with 50% overlap. Two successive sub-windows were correlated using the ‘mqd’ method and aberrant vectors were filtered out using at a median filter and by imposing maximum limits on x and y velocity components. The time between successive frames was 2 min. Further analysis of the velocity field was performed with custom MATLAB code for calculating speed and directionality distributions, spatiotemporal heat plots, and absolute magnitude of average speed, x-velocity and directionality quantities. The Spatiotemporal heat plots were constructed by calculating the average speed from each interrogation windows running parallel to the epithelial edge (y-direction) and thereby creating an average speed profile perpendicular to the edge (x-direction) at each instance in time yield information about the location of the edge and average speeds within the sheet. All data represent analysis of the 3–12 hr time frame (9 hr) of the 18 hr time-lapse recording. The coefficient of variation (CV) is calculated by taking the ratio between the standard deviation and the mean of the angles defining the direction of cell sheet motion.

### Analysis of Cell Proliferation

Cell proliferation was determined using the acquired time-lapse images for each cell line under migration assay conditions and final results represent the average of three independent (separate days) experiments. For each image set, three stationary interrogation windows (169×169 µm or 2.86×10^−4^ cm^2^) were constructed parallel to the cell-sheet front and evenly spaced across the sheet. The number of mitotic events within each interrogation window was manually counted as the cells migrated over a 12 h period, and reported as the average number of mitotic events ⋅ cm^−2^ h^−1^.

### Western Blotting

Cells were cultured on collagen coated 6-well tissue culture dishes over night and the starvation / stimulation protocol described for the migration assay was applied. 6 h after stimulation with growth factors cells were lysed in RIPA lysis buffer (Sigma Aldrich, St. Louis, MO) supplemented with Complete Mini and PhosStop (Roche, Indianapolis, IN) to inhibit protein degradation and phosphatase action. Lysates were cleared by centrifugation (12,000 g, 10 minutes, 4°C), and protein concentrations of the supernatants determined by Bradford assay (BioRad, Hercules, CA). Proteins were then separated by sodium dodecyl sulfate-polyacrylamide gel electrophoresis (SDS-PAGE) and transferred to PVDF membranes. Membranes were blocked with 3% bovine serum albumin (fraction V, Biorad) in TBST (50 mM Tris pH 7.4, 0.05% Tween 20), probed with primary antibodies (anti-phosphoAkt [Ser 473] 1∶1000, anti-AKT 1∶1000, anti-EGFR 1∶1000, anti-phosphoERK [Thr202/Tyr204] 1∶1000, anti-ERK 1∶1000 (all Cell Signaling Technology, Danvers, MA), anti-E-cadherin 1∶5000 (Invitrogen), or anti-actin 1∶60,000 (Chemicon)) followed by incubation with a horseradish peroxidase (HRP) conjugated antibody (anti-rabbit IgG∼HRP, 1∶5000 or anti-mouse IgG ∼ HRP 1∶5000 (Pierce, Rockford, IL)). Antigens were visualized by enhanced chemiluminescence (Amersham, GE Healthcare, Pittsburg, PA).

### LPA Receptor Gene expression analysis

RNA from each cell line was purified for gene expression analysis using RNeasy® kit (Qiagen, Valencia, CA). The quality and integrity of the RNA was analyzed on an Agilent BioAnalyzer 3000. Total cellular RNA samples with a RIN >9 were used for further microarray analysis. 100 ng of RNA was reverse transcribed and amplified using Ambion WT expression kit and sense strand cDNA was fragmented and biotynylated using Affymetrix WT terminal labeling kit following manufacture's instructions. Three biological replicates for each cell lines were hybridized to the Affymetrix GeneChip Human ST 1.0 at 45°C, 60 rpm for 16 hrs. After hybridization, Washing and staining were performed on an Affymetrix Fluidics Station 450 s using the Affymetrix GeneChIP hybridization Wash and Stain kit. Gebechips were scanned on an Affymetrix GeneChIP scanner 3000 7G and the data was collected using Affymetrix AGCC software. Gene expression datasets were normalized by RMA method using Affymetrix Expression Console and then analyzed using Partek Genomic Suite 6.5. (Partek, St. Louis, MO).

### Immunofluorescence

Using the same experimental setup for the unconstrained migration assay described in the Materials and Methods, M1–M4 cell lines were cultured in basal medium, 5 ng/mL EGF, or 1 µM LPA. After 6 hrs of treatment, cells were fixed with 4% paraformaldehyde in serum free culture medium. Autofluorescence was quenched with 0.1 M glycine, and cells were permeabilized with 1% saponin (Sigma), followed by blocking unspecific protein binding with 1% BSA in DPBS. Specimens were incubated with primary antibody in 1% BSA/DPBS (anti-E-Cadherin, 1∶800 [Invitrogen]; anti vimentin, 1∶100 [Daiko] at 4°C overnight, followed by an incubation with anti-mouse IgG or anti-rabbit IgG antibodies that were conjugated to Alexa 488 or Alexa 568 (dilution: 1∶500 or 1∶250, respectively, all Invitrogen). Nuclei were labeled with DAPI, and specimens were mounted using ImmuMount (Thermo Scientific). Specimens were then imaged using a Zeiss Observer.Z1 inverse microscope.

## Supporting Information

Figure S1 **Transwell and unconstrained migration assay.** (**A**) The number of M1–M4 cells (DAPI stained nuclei) that migrated through collagen IV coated transwell membranes after 4 hrs was measured by fluorescence microscopy. Data represent the mean ± SD of 3 independent experiments (see Materials and Methods). (**B**) Schematic of the experimental timeline for the unconstrained migration assay and when cell lysates were collected for western blot analyses (Fig S3A).
(TIFF)

Figure S2 **EGF and LPA have no impact on mitotic events in M1–M4 cells.** (**A**) Number of mitotic events counted during migration (see Material and Methods). (**B**) Graph depicting the average of horizontal speed components (V_x_), which mirrors the net displacement, for M1–M4 cells. (**C**) The average rate of mitotic events during the course of the migration experiment for M1–M4 cells under control (basal media), 5 ng/mL EGF and 1 µM LPA treatments. * p<0.05, ** p<0.01, *** p<0.001 (Tukey-Kramer test, n = 3 for all conditions except M2 with EGF where n = 2). All bar graphs report the mean ± SD.
(TIFF)

Figure S3 **Expression of key signaling components in M1–M4 cells.** (**A**) Representative Western blot result (n = 3) showing the expression of key signaling components in M1–M4 cells under the indicated treatment conditions. (**B**) Microarray analysis of RNA isolated from each cell line in the MCF10A series indicates that all four cell lines express comparable levels of mRNA for each of the LPA receptors tested. Gene expression datasets were normalized by RMA method using Affymetrix Expression Console and then analyzed using Partek Genomic Suite 6.5. (Partek, St. Louis, MO). Data from 4 independent experiments reported as mean ± SD.
(TIFF)

Figure S4 **Spatiotemporal directionality heat plots of M4 and MDA-MB 231T cells.** Spatiotemporal directionality plots were generated using PIV measurements as described. (**A**) 10 µM LPAR1 and 3 antagonist Kil6425 or DMSO (vehicle control) was added to M4 cells 20 min before stimulation of M4 cells with LPA (1 µM). Cells were allowed to migrate for 18 hrs. (**B**) The spatiotemporal directionality profiles of MDA-MB 231T cells are presented for control, 5 ng/mL EGF and 1 µM LPA treatment conditions. Data show representative heat plots from 3 independent experiments.
(TIFF)

Figure S5 **Directionality is a promising indicator of tumorigenic potential.** Combined migration data collected from the M1–M4 and MDA-MB-231T cells under basal conditions. Data sets depict mean ±95% CI of each metric and individual data points represent independent experiments. No correlation with tumorigenic potential is observed when comparing (**A**) migration distance or (**B**) average cell speed. (**C**) Tumorigenic cell lines (M3, M4 and 231T) harbor less directed motions (higher CVs) compared to more benign cell lines (M1 and M2). Statistical significance: * p<0.05, ** p<0.01, *** p<0.001 (Tukey-Kramer test, n = 6–7). All comparisons were made with M1 cells unless indicated by pairing-brackets.
(TIFF)

Figure S6 **Immunofluorescence shows altered E-cadherin profiles in MCF10A series.** Expression of E-cadherin was visualized by immunofluorescence 6 h after stimulation of cells with 0.1% horse serum (control), 5 ng/ml EGF, or 1 µM LPA. DAPI was used to label cell nuclei.
(TIFF)

Figure S7 **E-cadherin protein expression is reduced in M3 and M4 cells.** Representative Western blot result (n = 3) showing the expression of E-cadherin in M1–M4 cells under the indicated treatment conditions.
(TIFF)

Figure S8 **Immunofluorescence shows vimentin profiles unchanged in MCF10A series.** Expression of vimentin was visualized by immunofluorescence 6 h after stimulation of cells with 0.1% horse serum (control), 5 ng/ml EGF, or 1 µM LPA. DAPI was used to label cell nuclei.
(TIFF)

Movie S1 **M1 cells migration into open space under control conditions.** Phase images were taken every 2 min for 12 hrs. Playback is 35× normal and scale bar  = 100 µm.
(MOV)

Movie S2 **M2 cells migration into open space under control conditions.** Phase images were taken every 2 min for 12 hrs. Playback is 35× normal and scale bar  = 100 µm.
(MOV)

Movie S3 **M3 cells migration into open space under control conditions.** Phase images were taken every 2 min for 12 hrs. Playback is 35× normal and scale bar  = 100 µm.
(MOV)

Movie S4 **M4 cells migration into open space under control conditions.** Phase images were taken every 2 min for 12 hrs. Playback is 35× normal and scale bar  = 100 µm.
(MOV)

Movie S5 **During uniform LPA (1 µM) stimulation, lung, colony forming M4 cells exhibit migration behavior similar to the non-transformed breast epithelial cell line, M1.** Phase images were taken every 2 min for 12 hrs. Playback is 35× normal and scale bar  = 100 µm.
(MOV)
