# Supplementary figures and images for: Real-Time Motion Analysis Reveals Cell Directionality as an Indicator of Breast Cancer Progression
Source: PLoS One. 2013 Mar 19;8(3):e58859. doi: 10.1371/journal.pone.0058859 (PMC3602596; doi:10.1371/journal.pone.0058859)

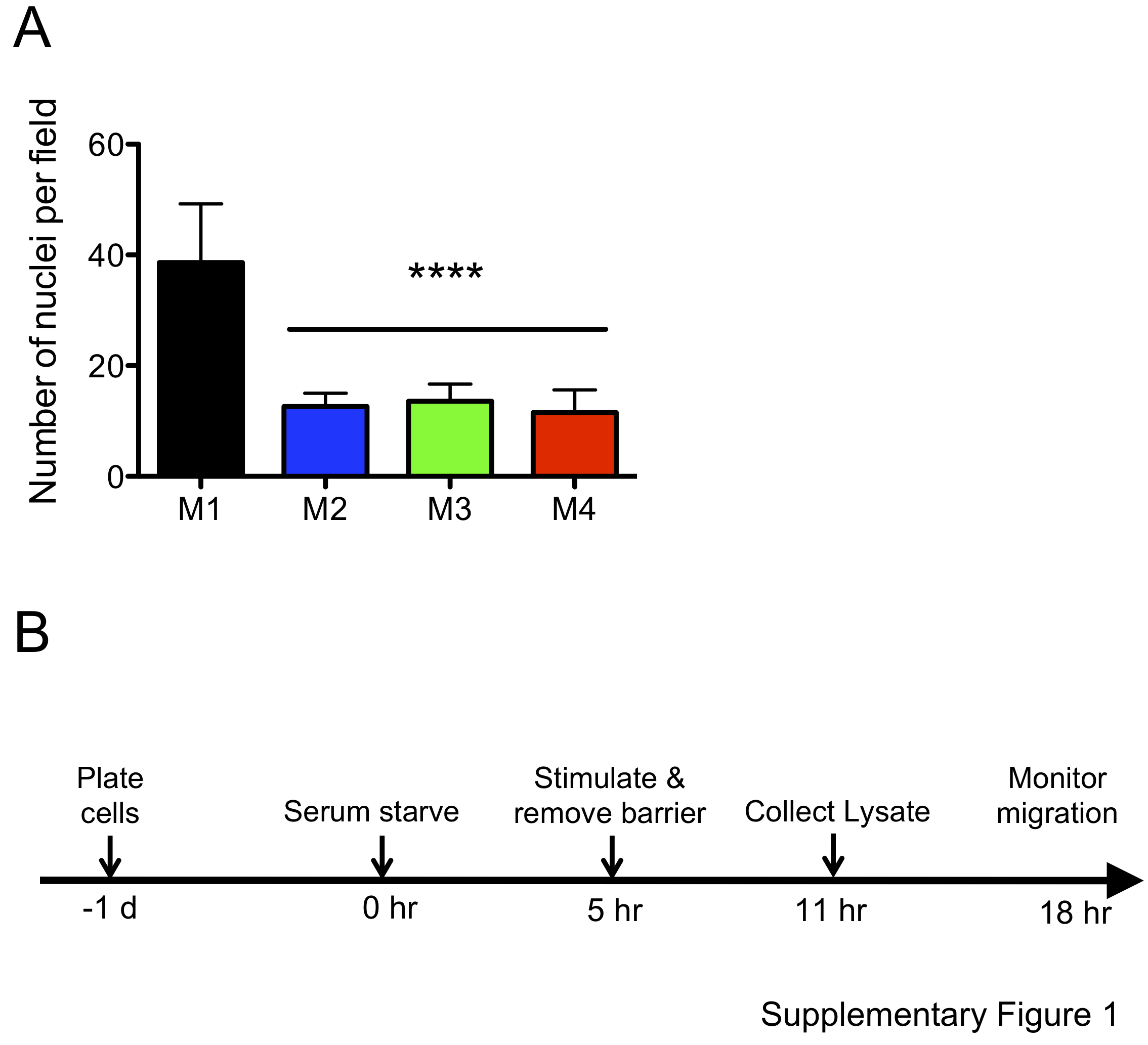

Supplement: Figure S1 — Transwell and unconstrained migration assay. (A) The number of M1–M4 cells (DAPI stained nuclei) that migrated through collagen IV coated transwell membranes after 4 hrs was measured by fluorescence microscopy. Data represent the mean ± SD of 3 independent experiments (see Materials and Methods). (B) Schematic of the experimental timeline for the unconstrained migration assay and when cell lysates were collected for western blot analyses (Fig S3A). (TIFF) [file pone.0058859.s001.tif]

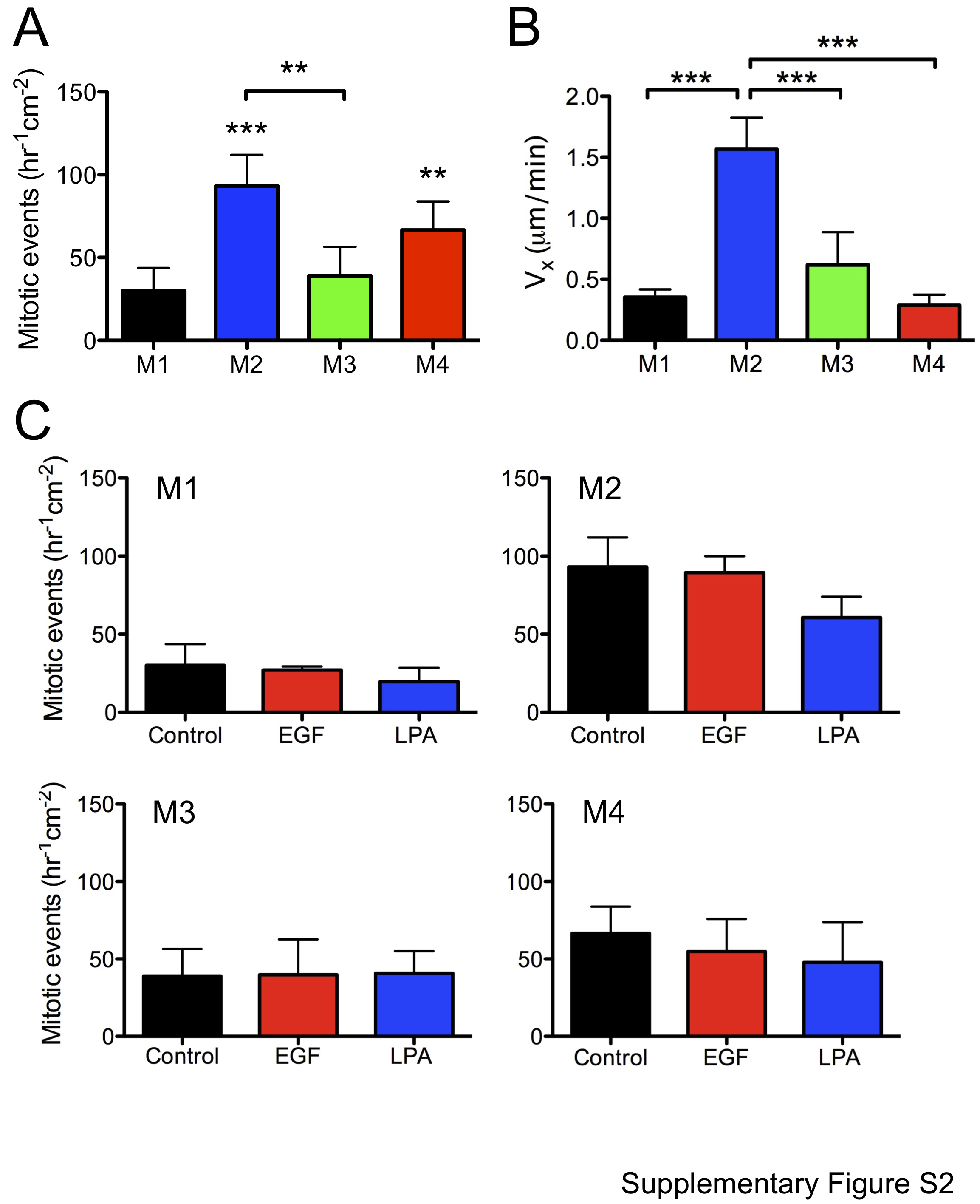

Supplement: Figure S2 — EGF and LPA have no impact on mitotic events in M1–M4 cells. (A) Number of mitotic events counted during migration (see Material and Methods). (B) Graph depicting the average of horizontal speed components (Vx), which mirrors the net displacement, for M1–M4 cells. (C) The average rate of mitotic events during the course of the migration experiment for M1–M4 cells under control (basal media), 5 ng/mL EGF and 1 µM LPA treatments. * p<0.05, ** p<0.01, *** p<0.001 (Tukey-Kramer test, n = 3 for all conditions except M2 with EGF where n = 2). All bar graphs report the mean ± SD. (TIFF) [file pone.0058859.s002.tif]

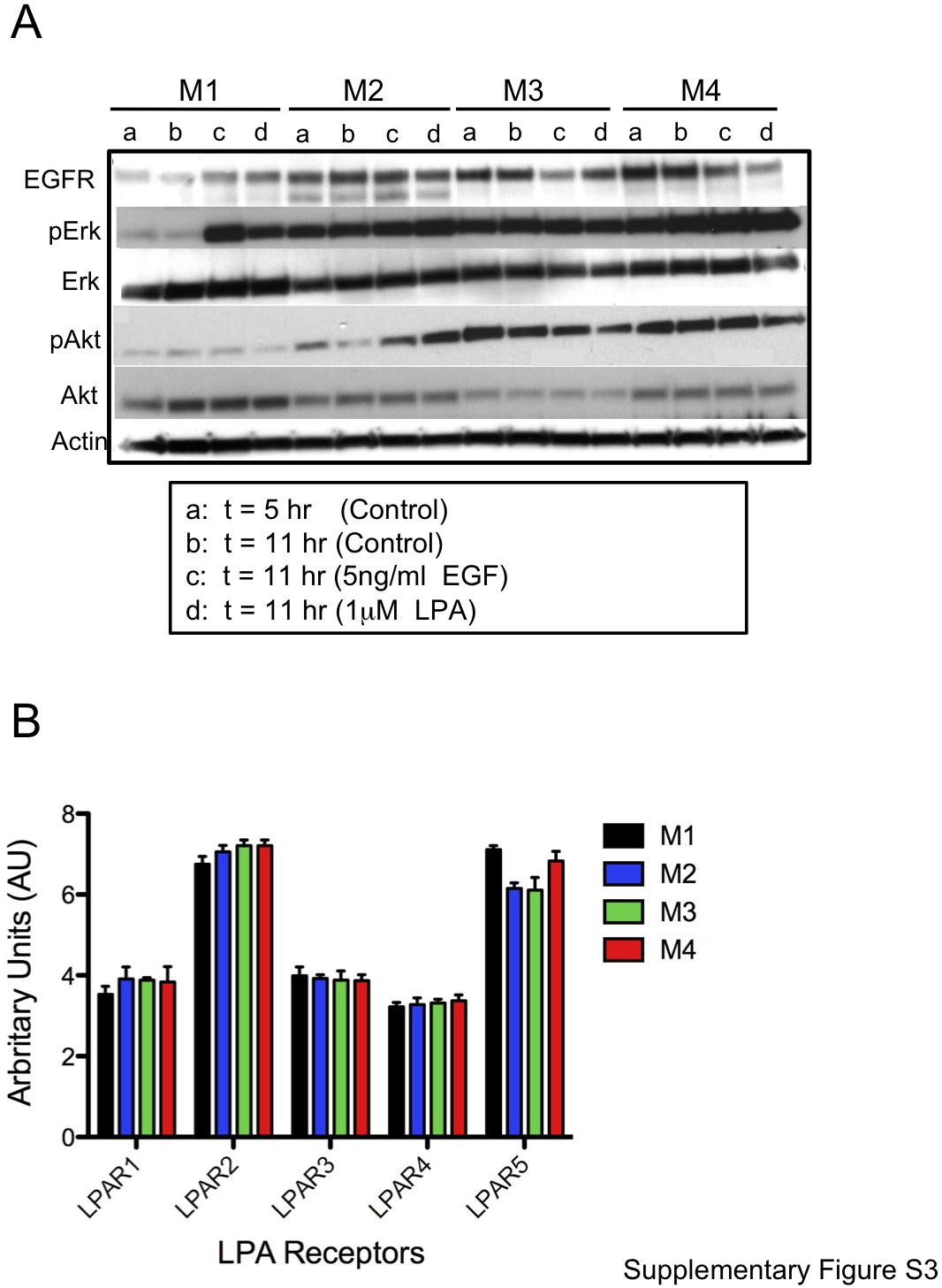

Supplement: Figure S3 — Expression of key signaling components in M1–M4 cells. (A) Representative Western blot result (n = 3) showing the expression of key signaling components in M1–M4 cells under the indicated treatment conditions. (B) Microarray analysis of RNA isolated from each cell line in the MCF10A series indicates that all four cell lines express comparable levels of mRNA for each of the LPA receptors tested. Gene expression datasets were normalized by RMA method using Affymetrix Expression Console and then analyzed using Partek Genomic Suite 6.5. (Partek, St. Louis, MO). Data from 4 independent experiments reported as mean ± SD. (TIFF) [file pone.0058859.s003.tif]

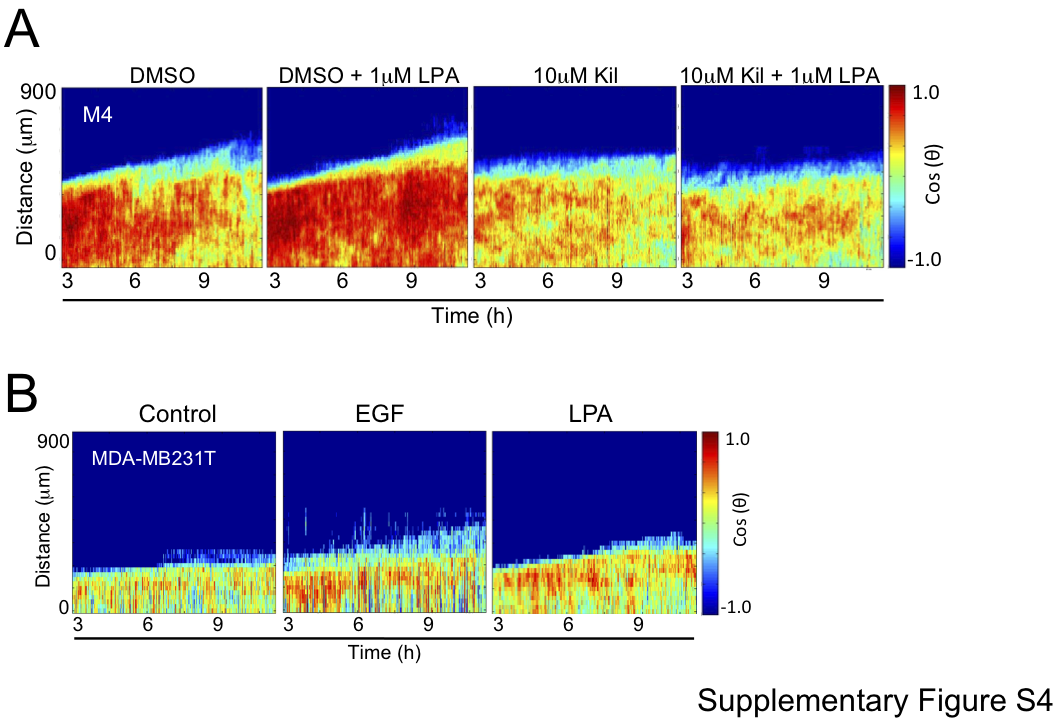

Supplement: Figure S4 — Spatiotemporal directionality heat plots of M4 and MDA-MB 231T cells. Spatiotemporal directionality plots were generated using PIV measurements as described. (A) 10 µM LPAR1 and 3 antagonist Kil6425 or DMSO (vehicle control) was added to M4 cells 20 min before stimulation of M4 cells with LPA (1 µM). Cells were allowed to migrate for 18 hrs. (B) The spatiotemporal directionality profiles of MDA-MB 231T cells are presented for control, 5 ng/mL EGF and 1 µM LPA treatment conditions. Data show representative heat plots from 3 independent experiments. (TIFF) [file pone.0058859.s004.tif]

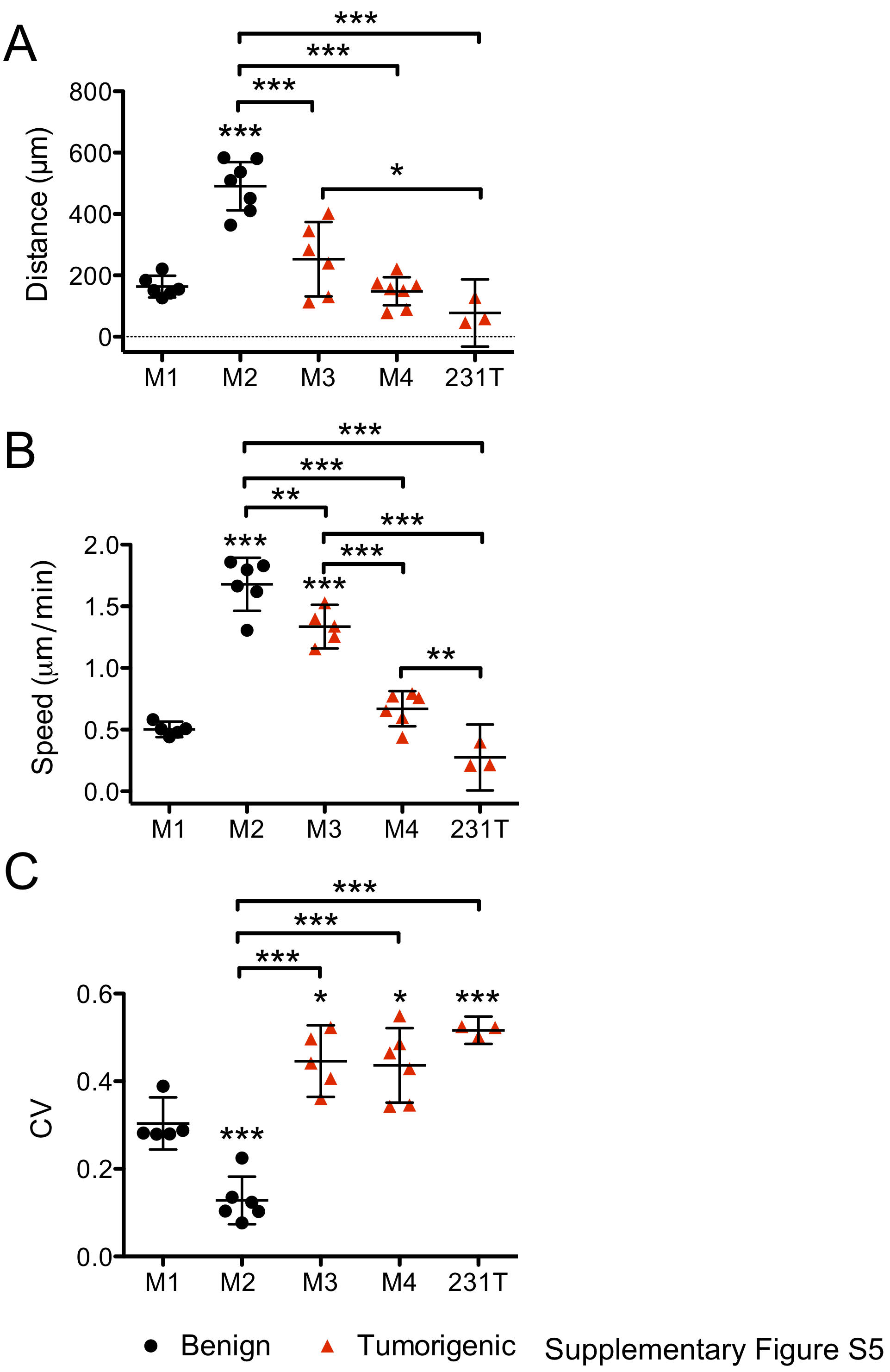

Supplement: Figure S5 — Directionality is a promising indicator of tumorigenic potential. Combined migration data collected from the M1–M4 and MDA-MB-231T cells under basal conditions. Data sets depict mean ±95% CI of each metric and individual data points represent independent experiments. No correlation with tumorigenic potential is observed when comparing (A) migration distance or (B) average cell speed. (C) Tumorigenic cell lines (M3, M4 and 231T) harbor less directed motions (higher CVs) compared to more benign cell lines (M1 and M2). Statistical significance: * p<0.05, ** p<0.01, *** p<0.001 (Tukey-Kramer test, n = 6–7). All comparisons were made with M1 cells unless indicated by pairing-brackets. (TIFF) [file pone.0058859.s005.tif]

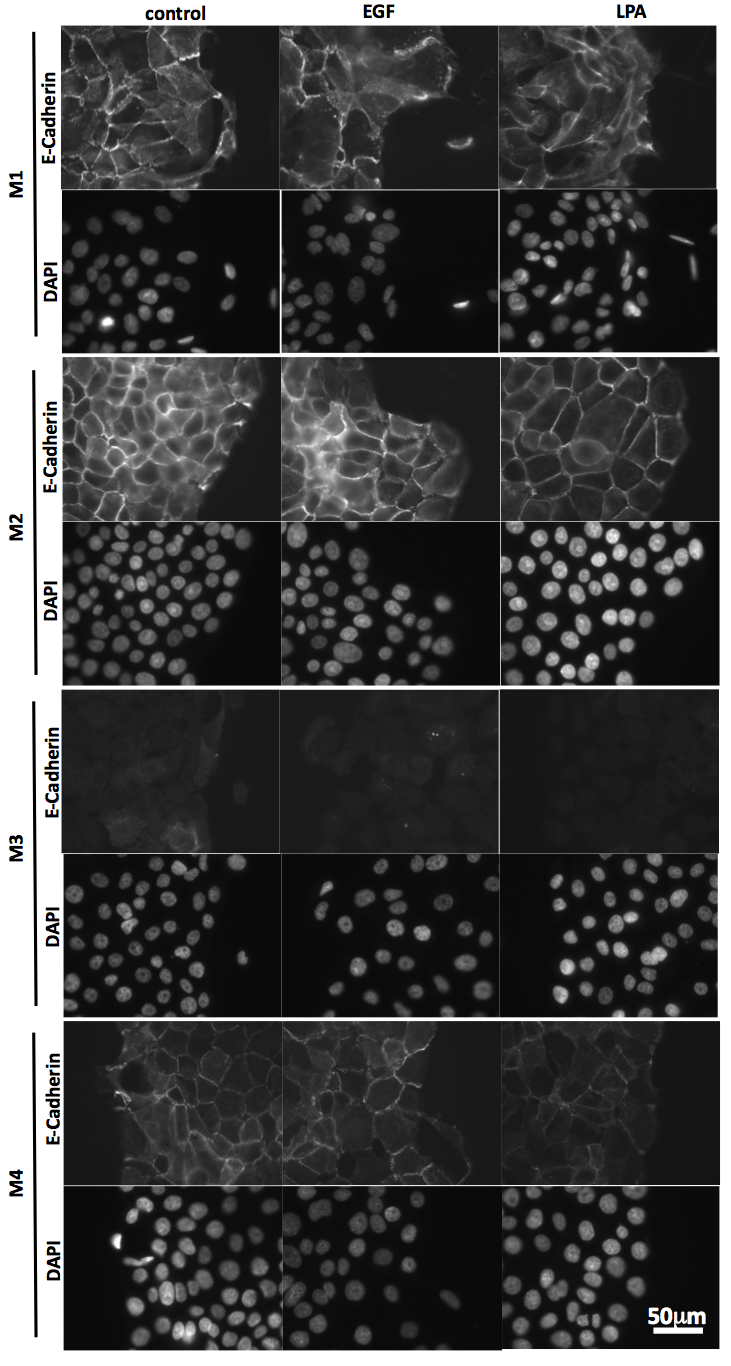

Supplement: Figure S6 — Immunofluorescence shows altered E-cadherin profiles in MCF10A series. Expression of E-cadherin was visualized by immunofluorescence 6 h after stimulation of cells with 0.1% horse serum (control), 5 ng/ml EGF, or 1 µM LPA. DAPI was used to label cell nuclei. (TIFF) [file pone.0058859.s006.tif]

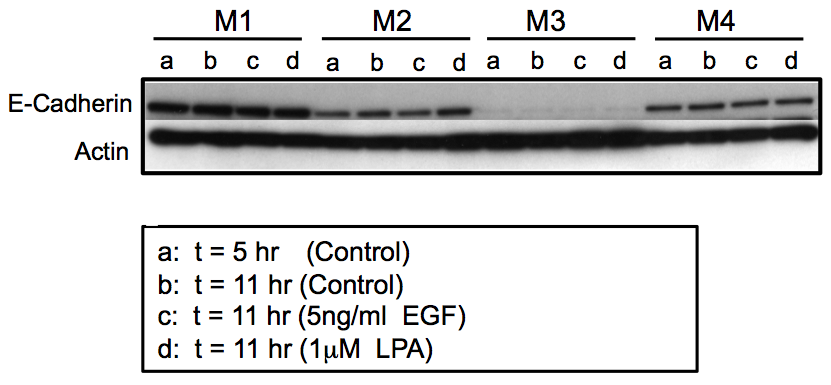

Supplement: Figure S7 — E-cadherin protein expression is reduced in M3 and M4 cells. Representative Western blot result (n = 3) showing the expression of E-cadherin in M1–M4 cells under the indicated treatment conditions. (TIFF) [file pone.0058859.s007.tif]

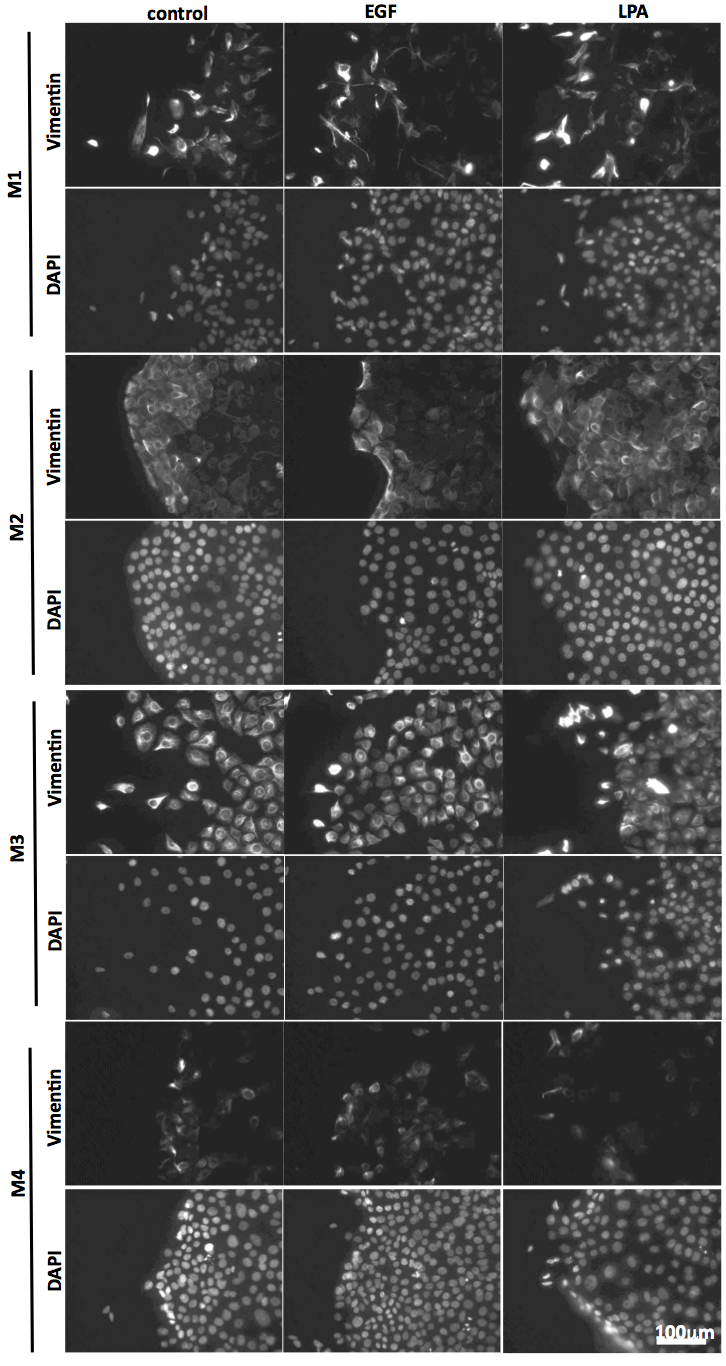

Supplement: Figure S8 — Immunofluorescence shows vimentin profiles unchanged in MCF10A series. Expression of vimentin was visualized by immunofluorescence 6 h after stimulation of cells with 0.1% horse serum (control), 5 ng/ml EGF, or 1 µM LPA. DAPI was used to label cell nuclei. (TIFF) [file pone.0058859.s008.tif]
